# Shape Evolution of MgO Nanocubes via Rapid pH Self-Adjustment at Oxide–Water Interfaces

**DOI:** 10.1021/acsnanoscienceau.6c00030

**Published:** 2026-05-15

**Authors:** Romane Cavrot, Aminata Diouf, Cedric Baumier, Jacek Goniakowski, Livia Giordano, Slavica Stankic

**Affiliations:** † Institut des NanoSciences de Paris (INSP), Sorbonne Université, CNRS UMR 7588, 4 place Jussieu, 75252 Paris Cedex 05, France; ‡ Laboratory of the Physics of the two Infinities Irène Joliot-Curie (IJCLab), Université Paris-Saclay, CNRS UMR 9012, Orsay Cedex 91405, France; § Department of Materials Science, Università degli Studi di Milano-Bicocca, Via Cozzi 55, Milano 20125, Italy

**Keywords:** MgO nanopowder, pH, shape transformation, defects, surface energies, oxide–water interface

## Abstract

Magnesium oxide (MgO) is a prototypical model for oxide–water interfaces and a technologically relevant material in catalysis, environmental remediation, and corrosion-resistant coatings. Here, the dissolution behavior and morphology evolution of MgO smoke nanocubes are investigated in aqueous media while simultaneously monitoring the self-adjusting solution pH under three initial conditions: distilled water (pH ≈ 6.4), mildly acidic HCl solution (pH = 6), and alkaline NaOH solution (pH = 11). Time-resolved TEM reveals that dissolution initiates preferentially at (110) edges, drives a progressive transition from cubic to truncated cubes and finally octahedral morphologies exposing (111) facets, and is accompanied by brucite [Mg­(OH)_2_] formation whose extent depends on particle size and pH. Upon immersion in distilled water or a mildly acidic solution, the suspension pH rapidly rises to ≈11–12 within minutes, whereas this abrupt pH change is not observed when MgO is introduced directly into an already alkaline medium (pH 11). A subsequent quasi-stationary regime with pH maintained at 11–12 persists for days, showing that the vast majority of the observed morphological transformations in these systems actually proceed under strongly alkaline rather than nominally neutral or mildly acidic conditions. To rationalize these observations, density functional theory calculations are performed for ideal and defect-containing low-index MgO surfaces under H^+^-rich conditions, including protonated Mg-vacancy (V_Mg_H_2_) defects relevant to nonoxidative dissolution. The calculations reveal a strong pH dependence of defective surface energies, pronounced anisotropy in the thermodynamic driving force for dissolution, and higher Mg^2+^ extraction barriers for compact (100) than for more open (110) and (111) terminations, consistent with the experimentally observed facet-dependent kinetics. These results present a unified, pH-dependent picture of MgO dissolution and morphology evolution in water, clarify that earlier “neutral” dissolution studies actually probed alkaline conditions, and offer guidelines for controlling MgO stability and Mg­(OH)_2_ formation in aqueous media. They highlight how self-adjusted local pH and defect chemistry regulate dissolution, shape transformation, and passivation of oxide nanomaterials at solid–liquid interfaces relevant to environmental and energy-related (electro)­chemical systems.

## Graphical abstract

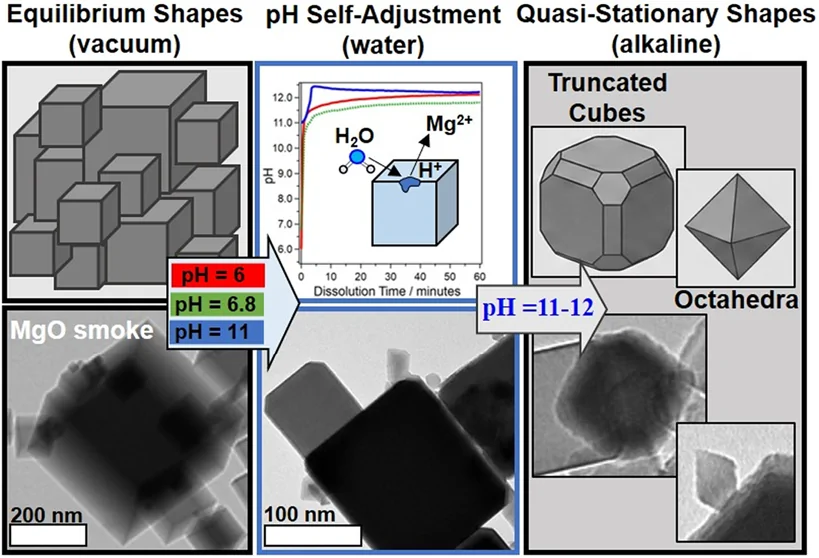

## Introduction

The interaction of liquid water with solid materials encompasses wetting, adsorption, dissolution, and interfacial structuring—processes central to biology, materials science, (electro)­catalysis, and environmental chemistry.^1–3^ In surface science and (electro)­catalysis, water–solid interactions modulate adsorption energies, surface restructuring, adsorbate solvation, and reactivity, and mediate processes such as oxidation, dissolution, and proton-coupled electron transfer. Water’s ability to dissociate and form hydrogen-bonded networks at oxide surfaces strongly influences catalytic activity,^4–7^ whereas interfacial water plays a decisive role in corrosion,^8–11^ etching^12^ and nanoparticle growth.^13^ These broad implications underscore the need to understand oxide–water interfaces under realistic aqueous conditions.

Magnesium oxide is a prototypical system for elucidating water–oxide interactions and a versatile oxide nanomaterial platform for studying how particle size, facet orientation, and defect chemistry govern dissolution, hydration, and morphology evolution at solid–liquid interfaces. It has been used for studying the competition between molecular and dissociative adsorption, the kinetics of oxide dissolution, the hydration reaction from MgO to Mg­(OH)_2_, the morphology evolution of ionic crystals in aqueous environments, and the effect of solution composition on these processes.^14–18^ Aside these fundamental aspects, MgO hydration is also relevant for several applications, including catalysis,^19^ heat storage,^20^ and cement production.^21^ Because of this relevance, the interaction of MgO with water has received considerable attention. Although MgO is thermodynamically unstable in water, kinetic barriers, interfacial water structure, and the formation of passivating layers can markedly slow its dissolution and transformation into Mg­(OH)_2_ by stabilizing the oxide–water interface.^22,23^

The interaction of MgO powders consisting of cubes exposing MgO(100) facets with water vapor yields predominantly molecular adsorption, with only a minor fraction undergoing dissociation, mainly at defects such as undercoordinated sites (steps, edges, and corners), vacancies, and strained regions.^24–30^ This picture is confirmed by density functional theory calculations and scanning tunneling microscopy studies on MgO(100) surfaces, which show molecular adsorption on thick, bulk-like MgO films and water dissociation on ultrathin films supported on metals.^31–33^ Upon increasing water coverage on ideally flat MgO(100) surfaces, both experimental and theoretical studies consistently indicate partial dissociation within the first water layer due to the formation of hydrogen bonds between water molecules^34–37^ – culminating in the formation of (4 × 2) and (3 × 2) ordered monolayer structures on the MgO(100) surface^34,38–42^ – a hydration structure recently shown to reproduce vibrational sum frequency generation spectra under ambient conditions.^23,43^

The interaction of water with MgO strongly depends on surface orientation and thereby modifies particle shape.^44–46^ Compared to MgO(100), more open orientations such as (110) and (111) exhibit predominantly dissociative adsorption, leading to dense hydroxyl terminations.^17,47–50^ The polar MgO(111) surface, electrostatically unstable in its ideal Tasker type-III form,^51–53^ can be stabilized either through reconstruction (e.g., octopolar motifs)^27^ or by hydroxylation.^54–58^

Experimentally observed water-driven evolution from cubic to octahedral morphologies^44^ was confirmed by first-principles Wulff constructions^55,59,60^ which indicate that under water-rich conditions, fully hydroxylated Mg-terminated (111) surfaces become energetically competitive and can dominate equilibrium morphologies. Indeed, Hacquart and Jupille provided seminal evidence that cubic MgO smoke crystals transform into octahedra terminated by hydroxyl-stabilized (111) facets during dissolution in water, with initial etching of (110) edges preceding the emergence of (111) facets.^44^ Size-dependent dissolution studies indicate that nanocubes below ∼ 10 nm dissolve rapidly, forming Mg­(OH)_2_ nanosheets, whereas larger cubes (10–1000 nm) dissolve more slowly due to the formation of hydroxide layers that passivate the (100) and (110) planes.^22^ Corroborating this, Pettauer et al. showed that MgO-to-brucite conversion rates are primarily governed by specific surface area.^61^ Complementary computational work has addressed the interplay between Mg^2+^ dissolution, water dissociation, and surface hydroxylation on MgO(100), pointing toward a dissolution–precipitation mechanism of Mg­(OH)_2_ formation rather than a solid-state reaction.^62^ Finally, on MgO single crystals and thin films, it was likewise demonstrated that hydroxide films strongly inhibit dissolution of these two facets and that dissolution rates depend sensitively on pH.^63^

The MgO dissolution mechanism and kinetics have also been the subject of intensive investigation, both computationally and experimentally.^17,64,65^ Several studies have examined the pH dependence of MgO surface chemistry and its point of zero charge (PZC), which lies near pH 11–12.^16,23,66–68^ Recent vSFG measurements at the MgO(100)–water interface showed that dissolution processes and interfacial water structures in acidic media depend on hydrodynamic conditions—flowing versus static water—which control H^+^ depletion compensation.^69^ In another recent vSFG-based study on single crystals of different orientations exposed to heavy water over a wide pH range (2–13), MgO(100) and MgO(111) were found to possess similar points of zero charge near pH ≈ 12 but to exhibit distinct pH-dependent interfacial water responses, emphasizing that orientation-dependent polarity, termination, and hydroxylation are central to stabilizing the MgO–water interface and governing its reactivity.^23^ Collectively, these findings highlight the intricate interplay among particle size, morphology, facet termination, pH-dependent charging, and hydrodynamics in determining the stability of oxide nanoparticles in aqueous environments. Yet, it remains unclear how different nanocrystal’s facets dynamically respond to pH and dissolution, and how this response ultimately governs the evolution of its shape. Building on this foundation, the present study investigates how pH, dissolution time, and surface orientation jointly control MgO dissolution mechanisms in liquid water, with explicit consideration of particle size from nanometer to submicron scales. By combining time-resolved TEM with in situ pH monitoring we show that dissolution in distilled water or mildly acidic solutions rapidly self-adjusts to pH 11–12 and then enters a quasi-stationary regime, so that most morphology changes actually occur under strongly alkaline conditions. Density functional theory reveals a pronounced pH dependence of defective surface energies and facet-resolved Mg^2+^ extraction barriers, explaining the faster dissolution of (110)/(111) relative to (100) surfaces, thus providing a unified framework for the observed MgO morphological transformation and Mg­(OH)_2_ formation in aqueous environments.

## Experimental and Computational Methods

### Synthesis and Characterization

MgO smoke powders were prepared by burning magnesium foil (Goodfellow, 99.9% purity) in a glovebox. This method facilitates the formation of oxide particles at temperatures near equilibrium shape, yielding broadly dispersed particle sizes.^70^ Powders were collected on a glass plate positioned 10 cm above the combustion zone to capture particles immediately after formation. Samples were stored under high vacuum (*P* < 10^–6^ mbar) prior to analysis to prevent air contamination. Transmission electron microscopy (TEM) was performed using JEOL JEM-1011 (100 kV) equipped with a Gatan Orius camera, and a Tecnai G2 20 Twin (200 kV) with spatial resolution of 0.27 nm and scanning transmission resolution of 0.08 nm, equipped with a Gatan OneView 4k × 4k camera. A representative TEM image and particle size distribution are shown in Figure 1A.

**Figure 1:**
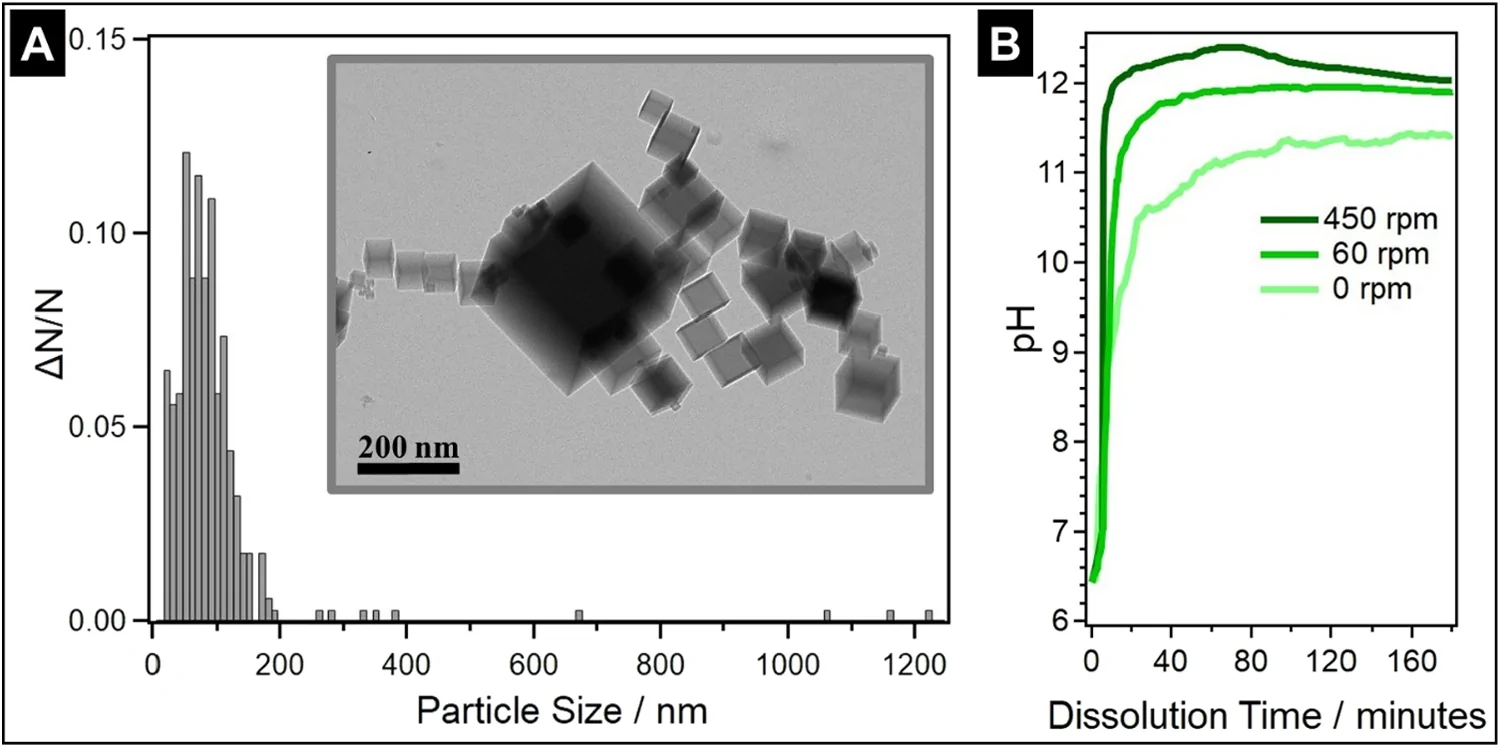
Particle size distribution of pristine MgO smoke (A) and pH evolution (of MgO/distilled water solution) as a function of stirring speed (B). Inset in A shows a representative TEM image.

### Dissolution

Suspensions were prepared by dispersing 25 mg of MgO smoke powder in 25 mL of distilled water, followed by gentle magnetic stirring at 60 rpm for predetermined durations (2, 10, 60, 180, 360, 600, 1440, and 2880 min). Samples were collected by centrifugation at each time point, washed with ethanol, and dried. Experiments were also conducted at pH 6 and pH 11 by adjusting solutions with HCl (1 M) or NaOH, respectively.

### pH Measurements

Solution pH was measured using a digital pH meter (Moineau Instruments 2230LM). Three different stirring speeds were tested and the pH evolution as a function of stirring speed is shown Figure 1B.

### DFT Approach

DFT calculations were carried out using the Vienna Ab-initio Simulation Package (VASP),^71,72^ the Projector Augmented Wave approximation^73,74^ to simulate the electron–core interaction, and a 400 eV energy cutoff in the expansion of Kohn–Sham orbitals on a plane-wave basis set. Magnesium, oxygen and hydrogen pseudopotentials provided by VASP were used. Spin-unrestricted calculations were performed using the PBEsol^75^ exchange-correlation functional, which provides reliable predictions of both surface and adsorption energies. The calculated bulk MgO lattice parameter (4.22 Å) agrees closely with the experimental value and the computed surface energies for the ideal (100), (110), and (111) facets (1.02, 2.33, and 2.40 Jm^–2^, respectively) are consistent with the experimental data and previously reported LDA results.^27^ Likewise, the surface hydroxylation energies under normal conditions (−0.39, −1.91, and −2.20 Jm^–2^ for the three facets) are in good agreement with the PW91 values reported in refs 55. and^59^. Atomic configurations were plotted using VESTA.^76^

### Structural Models

Facets of MgO particles were modeled using slabs comprising five, six, and seven atomic layers for the (100), (110), and (111) orientations, respectively. A (2 × 2) surface unit cell was used in all cases, with Brillouin zones sampled using (4 × 4), (3 × 4), and (4 × 4) Monkhorst–Pack k-point meshes for the respective surfaces. Surface defects (one per cell) and/or hydroxylation layers (four H_2_O molecules per cell) were placed on one side of the slab. A vacuum spacing exceeding 10 Å was applied, and dipole corrections were systematically included. Atomic coordinates were relaxed until residual forces were below 0.01 eV Å^–1^, while the bottom MgO layers were fixed at their bulk positions. For the (111) facet, asymmetric slabs were used, terminated with octopolar reconstruction^27^ to compensate surface polarity, following the approach adopted in previous studies.^55^ Pristine and defective terminations were modeled using this reconstruction, whereas fully hydroxylated ideal surfaces were represented by ideal bulk terminations saturated by OH^–^ and H^+^ species originating from water dissociation on the two sides of the slab. For this surface orientation, the reported energies correspond to averages over O- and Mg-terminated surfaces.

### Extraction Energies

Calculations of Mg^2+^ extraction from the H^+^-rich surface (two H^+^ per cell) were performed in a charged cell, allowing explicit account for the excess +2 charge. Because only energy differences computed within the very same unit cell were analyzed, most errors associated with long-range electrostatic interactions canceled out. In contrast, calculations of pristine and hydroxylated ideal as well as defective surfaces were carried out in neutral cells.

### Ab-Initio Thermodynamics

The thermodynamic considerations make use of the Computational Hydrogen Electrode,^77^ which assumes equilibrium between *H* ^+^+*e* ^–^ and 1/2 H_2(g)_ under standard conditions (pH = 0, zero potential versus the Standard Hydrogen Electrode (SHE))

1 $$2\mu \left(H_{\left(\mathrm{aq}\right)}^{+}\right)=-{2\mu (e}^{-})+\mu \left(H_{2\left(g\right)}\right)-2eU_{\mathrm{SHE}}-{2k}_{B}T\;\ln\left(10\right)\mathrm{pH}$$

Moreover, equilibrium between *Mg*_(*aq*)_^2+^ ions (of activity *a*_*Mg* ^2+^_) and Mg electrode under standard conditions (*Mg*_(*aq*)_^2+^ + 2*e* ^–^ ⇄ *Mg*_(*s*)_ at *U*_*SHE*_ = −2.4 *V*) was further assumed:

2 $${\mu (Mg}_{\left(\mathrm{aq}\right)}^{2+})=-2\mu \left(e^{-}\right)+{G(Mg}_{\left(s\right)})-2e\left(U_{\mathrm{SHE}}+2.4V\right)+k_{B}T\;\ln\;a_{{Mg}^{2+}}$$

As a consequence, the chemical potential difference between the charged species reads:

$$\Delta \mu ={\mu (\mathrm{Mg}}_{\left(\mathrm{aq}\right)}^{2+})-2\mu \left(H_{\mathrm{aq}}^{+}\right)={G(\mathrm{Mg}}_{\left(s\right)})-4.8\mathrm{eV}+k_{B}T\;\ln a_{{\mathrm{Mg}}^{2+}}-\mu \left(H_{2\left(g\right)}\right)+{2k}_{B}T\;\ln\left(10\right)\mathrm{pH}$$ 3

With *G­(Mg_(s)_)* denoting free energy of bulk Mg. In all calculations of Gibbs free energies *G = E – TS + ZPE*, entropic contributions (TS) were derived from thermodynamic data for gas-phase species,^78^ whereas zero-point energies (ZPE) were estimated from the vibrational analysis of free species and surface OH groups^30^ at 300 K and a water partial pressure of 0.035 atm was assumed, corresponding to the vapor pressure of water at 300 K.

## Results

The dissolution of MgO powder was experimentally monitored under three different acidity conditions, and the results are presented accordingly: in distilled water (pH ≈ 6.4), in an acidic medium with HCl (pH = 6), and in a basic medium with NaOH (pH = 11).

### Dissolution in Distilled H_2_O

Representative TEM images of MgO smoke nanoparticles immersed in distilled water (pH ∼ 6.4) for increasing durations are presented in Figure 2. Within the initial 2 to 60 min, particles maintain their characteristic cubic morphology (Figure 2A–C). Pristine nanocubes exhibit nearly perfect cubic morphology with strong polydispersity: 20–50 nm primary peak, secondary fraction >200 nm up to 1200 nm (Figure 1A). Corrosion initiates preferentially at particle edges, leading to the development of truncated (110) facets. This effect is more pronounced in smaller nanoparticles (<50 nm) as visible from the insets in Figures 2A–B. This shape is represented by the corresponding Wulff construction shown in Figure 2J. These morphological changes are consistent with those observed by Hacquart and Jupille,^44^ despite the substantially different dissolution time scale for MgO smoke nanopowder. In order to obtain a complete picture of the pH conditions experienced by the particles at any given time during the measurement, parallel pH measurements were performed. The importance of monitoring pH evolution was recently demonstrated for a series of MgO samples of different origins, all converging toward pH > 10 on a relatively short dissolution time scale.^61^ As shown in Figure 2M, the solution pH rises sharply from ∼6.4 (*t* = 0) to 10.5 within 1 min and reaches ∼12 after 10 min upon MgO immersion in distilled water, remaining stable thereafter – in line with results obtained in ref 68. The rapid pH increase upon MgO immersion reflects the coupling between dissolution and surface protonation equilibria. Since the PZC of MgO lies near pH 11–12, the observed pH evolution indicates that the suspension rapidly approaches the PZC regime, where neutral MgO terminations dominate and dissolution slows down. This behavior is consistent with a protonation–dissolution–neutralization sequence, in which MgO dissolution consumes H^+^ from solution and progressively shifts the interfacial chemistry toward lower reactivity. To assess the influence of mass transport on alkalization, pH was monitored at different stirring speeds (Figure 1B; section Methods). Increasing stirring accelerates the pH rise, indicating that hydrodynamic conditions affect solute redistribution. These results also suggest that local pH gradients may transiently develop near the MgO surface at early times. Unlike prior studies, we simultaneously monitored pH and morphology, showing that the morphological changes observed for t_diss_ > 1 min occur under alkaline conditions (pH ≈ 11–12), rather than under the nominally neutral conditions assumed in previous studies.^44,59^ By 60 min (Figure 2C), the (110) facet etching extends to larger particles (>50 nm). Notably, no morphologies characteristic of Mg­(OH)_2_ were observed within this period, consistent with previous reports indicating the absence of brucite formation under short dissolution durations (<2 h).^22^ Figures 2D–F show particles after immersion times of 3, 6, and 10 h, respectively. Smaller particles (≤20 nm) lose their cubic morphology, whereas particles larger than 40 nm largely retain their cubic shape. The appearance of Mg­(OH)_2_ formation is evident around 3 h (Figure 2E, green arrows), with shapeless deposits forming between MgO particles. This observation is consistent with progressive supersaturation of the suspension with respect to brucite and marks the transition toward the long-term quasi-stationary pH regime. Although the deposits are consistent with brucite formation, a thin interfacial overlayer cannot be fully excluded. In addition, small octahedral particles (<20 nm) are observed in the inset of Figure 2E; these exhibit angles of 70–72°, consistent with (111) facets predicted by Wulff constructions (Figure 2K) are observed. At 10 h (Figure 2F), particles smaller than 80 nm present spherical or octahedral shapes (green frames), while free brucite is observed both adjacent to and detached from MgO particles (green arrows). The prevalence of larger octahedral particles (≤40 nm) increases compared to 6 h samples. Figures 2G–I depict samples after 1, 2, and 3 days of immersion. After 1 day (Figure 2G), large cubic particles (≥100 nm) remain visible, but smaller ones lose their original morphology. Octahedral particles persist, corroborated by the Wulff model in Figure 2L. By 2 days (Figure 2H), the predominant phase is Mg­(OH)_2_, with only large particles (≥200 nm) retaining edge etching along (110) facets. Octahedral particles persist at comparable sizes (≥200 nm). After 3 days (Figure 2I), the sample consists entirely of Mg­(OH)_2_, with occasional large particles approximating 500 nm. Quantitative analysis of particle size and facet ratios over time is shown in Figure 3A-B. Figure 3A quantifies the gradual disappearance of smaller MgO particles over time, based on counting a total of 380 cubic (nano)­particles in TEM images. We notice that PSD at *t* = 2 min (Figure 3A) matches *t* = 0 (Figure 1A), confirming no significant dissolution occurs before self-alkalization completes. The ratio of the distances h_110_/h_100_, reflecting the relative heights of (110) and (100) crystal faces, decreases as dissolution proceeds due to preferential corrosion at edges and corners. For ideal cubic particles, this ratio equals √2≈1.41; lower values indicate deviation from ideal cubic morphology. Smaller particles (≤80 nm) show a rapid decrease and reach an initial plateau between ∼1 and 3 h, whereas larger particles (∼200 nm) plateau between 3 and 6 h. After 6 h, nanocubes smaller than 80 nm are fully dissolved and absent from analysis. A secondary plateau in h_110_/h_100_ is observed after ∼1 day for larger particles, indicating morphological equilibrium, with measured plateau values consistent with literature reports; e.g., 1.19 ± 0.02 reported by Geysermans et al.^59^

**Figure 2:**
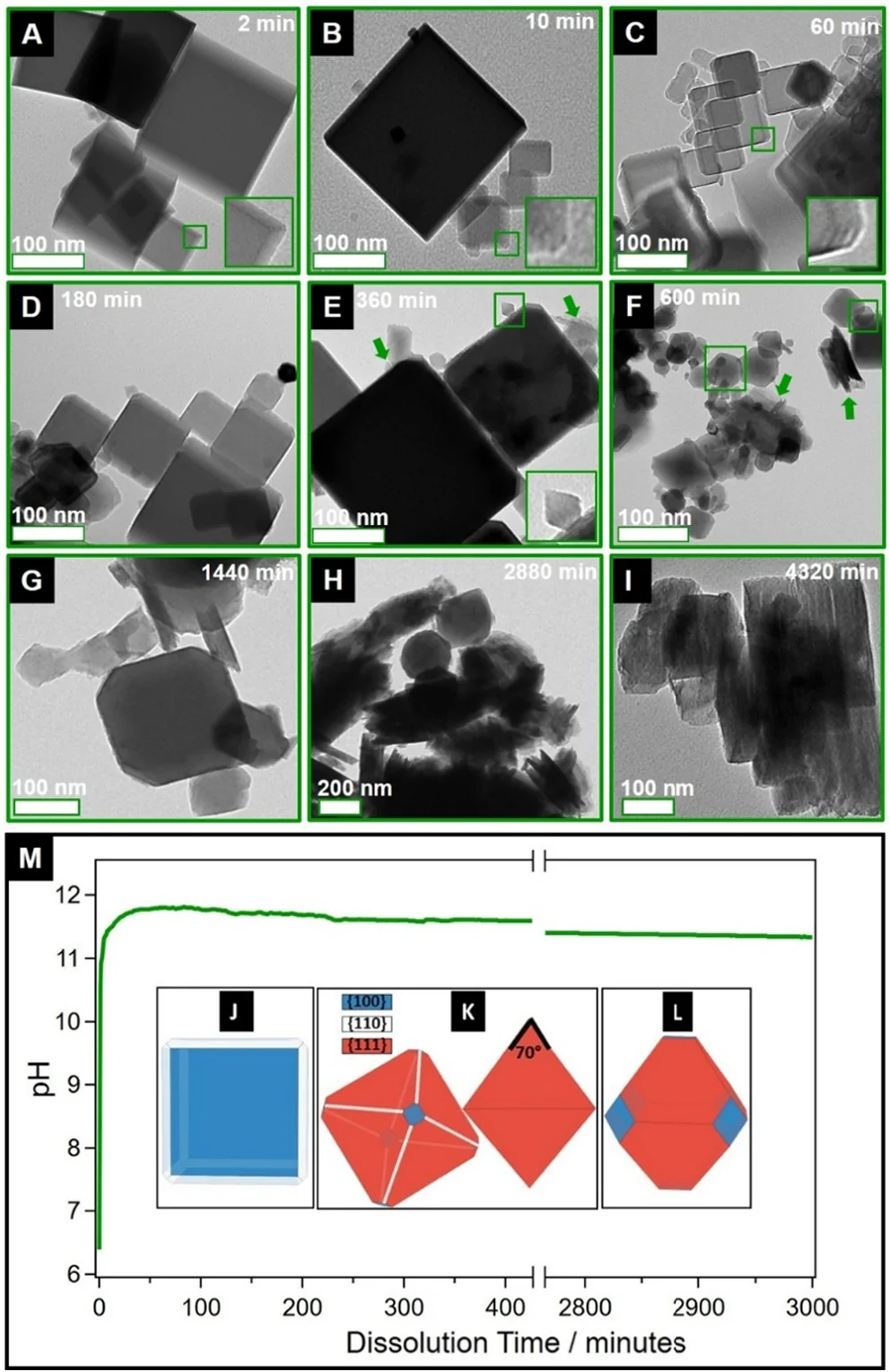
TEM images of MgO smoke particles immersed into distilled water for 2, 10, 60, 180, 360, 600, 1440, 2880, and 4320 min (A–I, respectively) and pH curve obtained as a function of time when MgO smoke powder was dissolved in distilled water (M). The presence of truncated cube and octahedra are shown in insets of (A–C) and (E), respectively, while the presence of brucite is indicated by arrows in (E) and (F). Corresponding particle shapes are shown in (J), (K), and (L).

**Figure 3:**
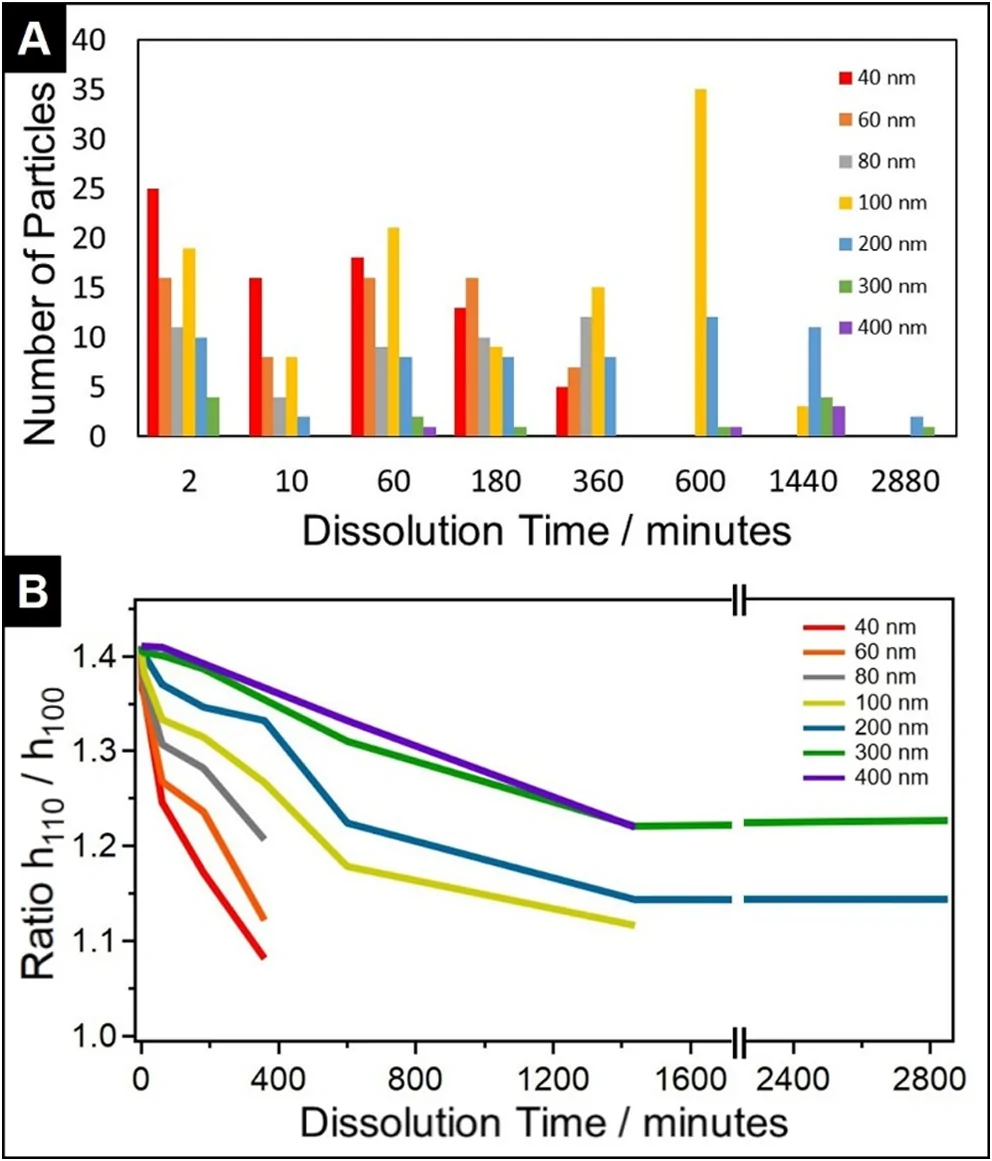
Time-dependent evolution of MgO particle size (A) and h110/h100 facet ratio (B) during aging in distilled water.

### Dissolution in Mildly Acidic Conditions

TEM images in Figure 4A show accelerated corrosion of MgO smoke nanoparticles in acidic conditions (pH 6) compared to distilled water. MgO nanocubes display pronounced attacks along (110) edges even in larger nanoparticles (∼80 nm) within 10 min (inset in Figure 4A) and the effect strengthen toward 60 and 180 min. The solution pH was also monitored over the full t_diss_ range under mildly acidic conditions. Red curve in Figure 4C shows that pH rises sharply from ∼6 (*t*_diss_ = 0) to 10.5 within 0.5 min and reaches ∼12 after 2 min (emphasized in inset of Figure 4C), demonstrating the strong buffering effect of MgO dissolution despite starting mildly acidic conditions. After the initial rapid pH variations under mildly acidic and neutral conditions, pH evolution slows markedly, converging to a single value—independent of the initial pH—over a much longer time scale (red and green curves, Figure 4C). This behavior is consistent with a protonation–dissolution–neutralization sequence, in which MgO dissolution consumes H^+^ from solution and progressively shifts the interfacial chemistry toward lower reactivity. By 60 min, nanoparticles below 30 nm lose cubic form (Figure 4A), with early octahedral shapes and Mg­(OH)_2_ deposits emerging. After 3 h (Figure 4A), attacks intensify and free Mg­(OH)_2_ precipitates – which was not observed at comparable times in distilled water. The h_110_/h_100_ ratios (Figure 4D) are consistently lower than in distilled water, with no clear plateau for 60–100 nm nanoparticles between 1–3 h. Smaller nanoparticles (∼40 nm) reach lower ratio values (∼1.095) than in water conditions (∼1.17), indicating more extensive morphological modification. Similar dissolution phenomena occur faster at pH 6, including particle size evolution, surface plane attacks, morphology changes, and Mg­(OH)_2_ formation.

**Figure 4:**
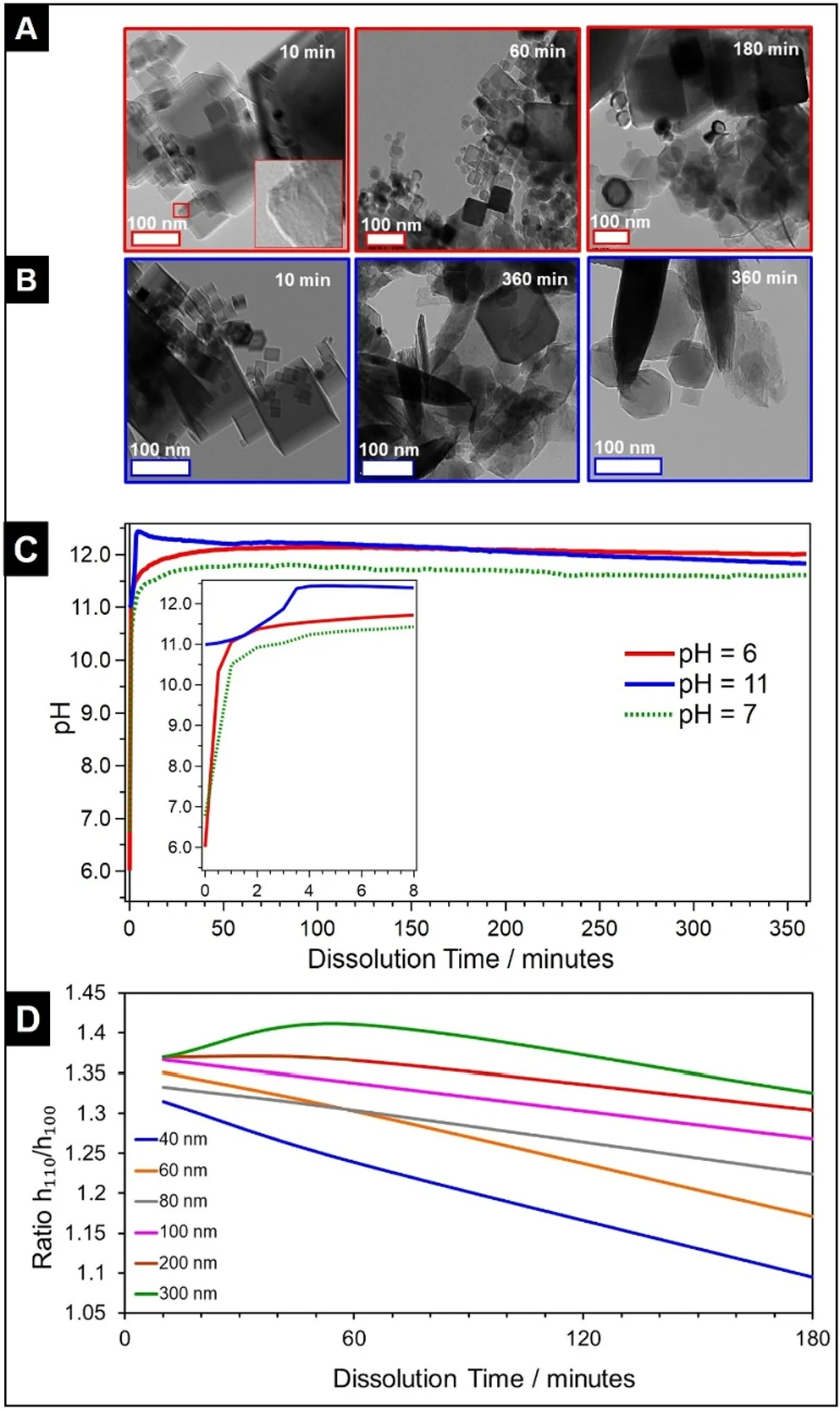
TEM images of MgO smoke particles immersed into: (A) mildly acidic solution (pH = 6) for 10, 60, and 180 min and (B) alkaline solution (pH = 11) for 10 and 360 min. Corresponding pH curves obtained as a function of time when MgO smoke powder was immersed in mildly acidic (red) and alkaline (blue) solutions are shown in (C). pH evolution in distilled water (green curve) versus time is added for comparison-. Inset highlights the sharp pH rise in red and green curves. Time evolution of the h_110_/h_100_ ratio during dissolution in mildly acidic solution for MgO (nano)­particles of different sizes (D).

### Dissolution in Basic Conditions

To test whether morphological changes differ when MgO is immersed directly into pre-equilibrated alkaline solution (pH 11)—as observed to develop rapidly under both neutral and mildly acidic starting conditions —dissolution was examined under initially high-pH conditions. Under basic conditions, the pH remains stable near 11.5 throughout the 6 h dissolution period (blue curve, Figure 4C), confirming strong solution buffering. MgO (nano)­particles largely retain cubic morphology with intact edges after 10 min whereas after 6 h, etching along (110) edges and corners appears, along with dissolution features on (111) planes for particles sized 40–100 nm (Figure 4B). At *t*_diss_ = 6h, Mg­(OH)_2_ precipitates are more abundant and larger (Figure 4B) compared to those observed in distilled water (Figure 2E).

Altogether, these observations demonstrate a dissolution process that initiates preferentially at MgO edges along (110) facets, follows by notable morphological evolution leading to octahedral morphologies exposing (111) facets and is accompanied by brucite formation. Dissolution in distilled or mildly acidic solutions rapidly self-adjusts to pH 11–12 and then enters a quasi-stationary regime, so that most observed morphology changes actually occur under strongly alkaline conditions.

## Theoretical Results

To gain insights into the origin of the substantial pH variations and the changes in particle shape and morphology observed when MgO particles are exposed to aqueous environments, we next investigated how pH affects the energetics of MgO (100), (110) and (111) surfaces identified by the experiment. Because the atomic-scale structures of these facets remain unresolved and are most likely governed by surface reaction kinetics, we employ ideal terminations that allow us to isolate the effect of the surface Mg cation coordination (5-, 4-, and 3-fold, respectively). This approach enables a direct assessment of how coordination dictates the orientation-dependent surface energetics.

### Ideal Terminations

As a reference, we considered ideal stoichiometric (100), (110), and (111) terminations, both pristine and fully hydroxylated, which have been extensively studied in the past and used to predict the morphology of MgO particles in dry and aqueous environments.^55,59^ Their key structural and energetic properties are summarized in Figures 5 and 6. Surface energies of hydroxylated terminations were obtained by adding Gibbs free energies of the hydroxylation reaction (Figure 5A1)

4 $$\Delta G\left(\mathrm{Mg}O_{\left(s\right)}+H_{2}O\to Mg_{\left(s\right)}OH^{-}+O_{\left(s\right)}H^{+}\right)=G\left(\mathrm{MgO}+H_{2}O\right)-G\left(\mathrm{MgO}\right)-G\left(H_{2}O\right)$$

to the energies of pristine terminations. In the above expression *G­(MgO)* and *G­(MgO+H*_2_ *O)* denote the calculated free energies of the pristine, and hydroxylated terminations, respectively, whereas *G­(H*_2_ *O)* is the free energy of isolated water molecules. Our calculations reproduce their well-established features, most notably the pronounced anisotropy of surface energies of the pristine terminations (with *E*(100) ≪ *E*(110) ≈ *E*(111), solid red lines in Figure 6A). This anisotropy arises primarily from the different coordination of surface ions—5-, 4-, and 3-fold for the (100), (110), and (111) terminations, respectively—as well as from the polar nature of the (111) surface. The substantially lower *E*(100) value accounts for the predominance of regular cubic MgO particles terminated by (100) planes in dry powders synthesized, for example, by combustion methods. Surface hydroxylation reverses this stability sequence (*E*(111-hydrox) < *E*(110-hydrox) < *E*(100-hydrox), dashed red lines in Figure 6A), since water more strongly interacts with more open or polar surfaces, where dissociative adsorption is highly favored (energy gain due to surface hydroxylation is indicated by the red shading), in contrast with the more compact (100) surface where only partial dissociation occurs. This inverted stability ranking of hydroxylated ideal MgO surfaces has been proposed to explain the octahedral, (111)-terminated morphologies observed for MgO particles under aqueous conditions. However, these considerations did not account for the solution pH, which abrupt variation represents one of key observations in the present experiments. Notably, the surface energies of ideal stoichiometric MgO surfaces—whether pristine or hydroxylated—as reported in the literature and reproduced in our present calculations, are independent of pH.

**Figure 5:**
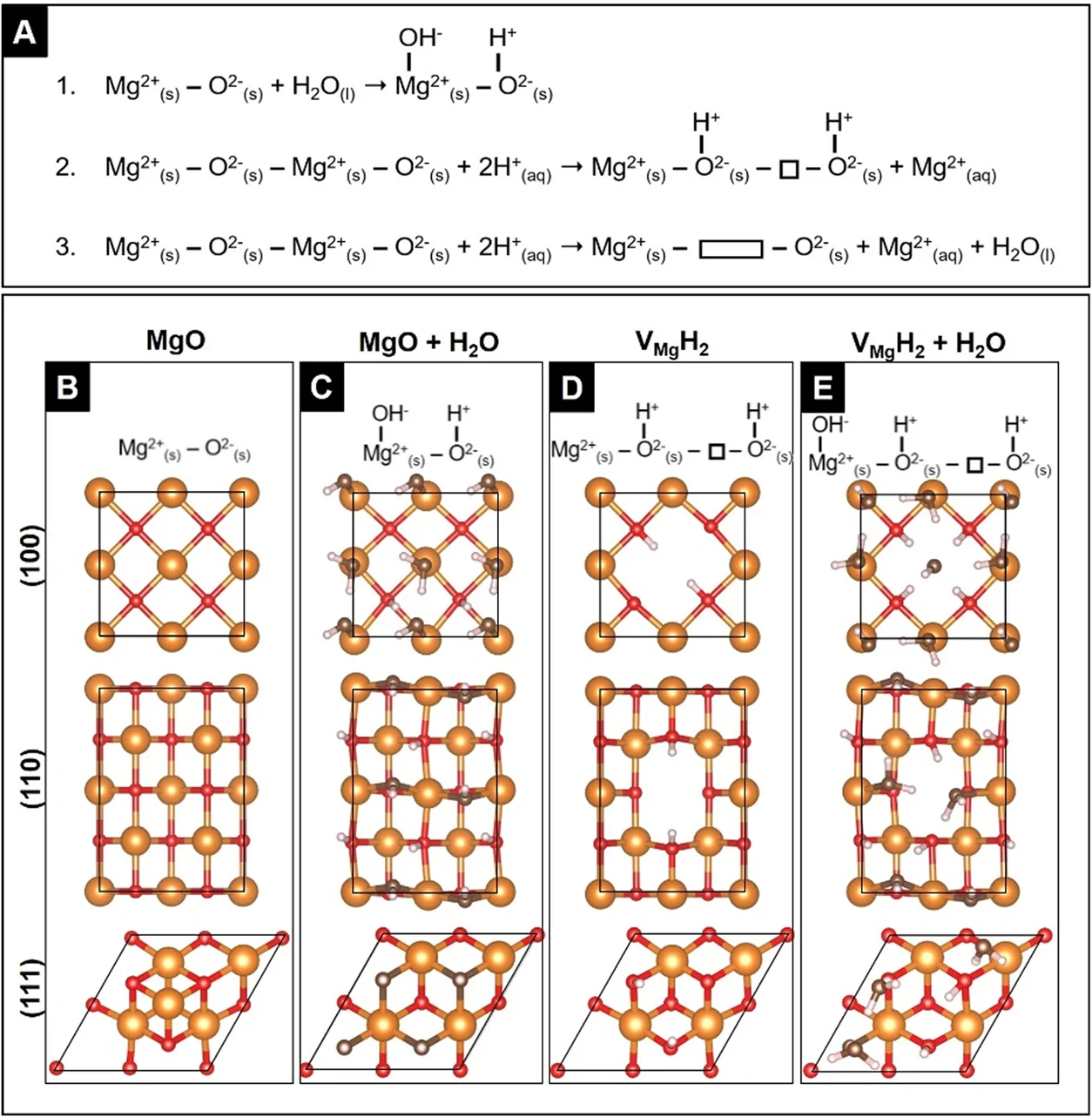
(A) Considered surface reactions. (B–E) Atomic configurations of ideal and defective (100), (110), and (111)-oriented MgO surfaces, with and without a hydroxylation layer. Color scheme: MgO surface oxygen (red spheres), magnesium ions (golden spheres), water-derived oxygen (brown spheres), and hydrogen atoms (white spheres).

**Figure 6:**
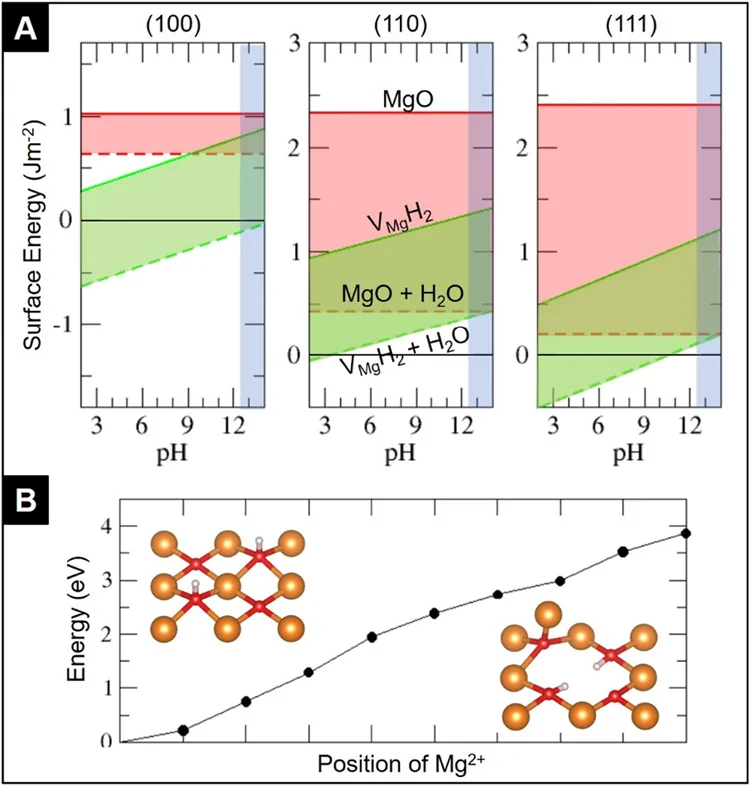
(A) Surface energies as a function of pH. Regions shaded in blue indicate calculated stability pH range of bulk brucite. (B) Energy profile of Mg^2+^ extraction from the (100) surface.

### Defective Terminations

To bridge this gap and incorporate factors directly related to the morphology changes observed in the present experiments, we studied the thermodynamics of surface dissolution by considering nonoxidative reactions. Such reactions, which do not involve any change in cation oxidation state, are common in minerals^79^ and are expected in high band gap materials composed of single-valent cations. First steps of a general nonoxidative dissolution reaction of MgO in H^+^-rich conditions (pH < PZC) involve the attack of H^+^ to surface oxygens, with subsequent leaching of Mg^2+^ into solution, which can be written as

5 $$\mathrm{Mg}O_{\left(s\right)}+2H_{\left(\mathrm{aq}\right)}^{+}\to {\mathrm{Mg}}_{\left(\mathrm{aq}\right)}^{2+}+H_{2}O$$

This reaction consumes H^+^, thus increasing the pH of the solution, and leads to the formation of a surface defect composed of an Mg vacancy and two surface hydroxyl groups (denoted V_Mg_H_2_, Figure 5D)

6 $$\Delta G\left(\mathrm{Mg}O_{\left(s\right)}+2H_{\left(\mathrm{aq}\right)}^{+}\to {V_{\mathrm{Mg}}H_{2\left(s\right)}+\mathrm{Mg}}_{\left(\mathrm{aq}\right)}^{2+}\right)=G\left(V_{\mathrm{Mg}}H_{2}\right)-G\left(\mathrm{MgO}\right)+\Delta \mu$$

In the above expression *G­(V*_*Mg*_ *H*_2_ *)* and *G­(MgO)* denote free energies of defective and ideal surfaces. We have verified that alternative surface reaction involving the formation of MgO vacancies V_MgO_, Figure 5A3, is systematically less favorable. Moreover, interaction of water with such surface MgO vacancies results in a spontaneous dissociation of H_2_O molecules and in oxygen filling the vacant lattice sites, thus leading to the same V_Mg_H_2_ defects.

We notice that in a broad range of acidic, neutral, and moderately alkaline pH, complete MgO dissolution is thermodynamically favored. The observed persistence of MgO particles reflects kinetic inhibition, with defect formation and coverage likely dictated by surface-reaction kinetics beyond the present scope. To maintain consistency, we applied a uniform defect coverage across the three MgO terminations and focused on the anisotropy of their calculated energetics, which provides insight into the thermodynamic bias underlying the experimentally observed orientation-dependent dissolution behavior.

### Effect of pH

As expected, the presence of excess H^+^ at the MgO surface leads to a strong pH dependence of the energies of V_Mg_H_2_-containing surfaces—both pristine (Figure 6A, solid green lines) and fully hydroxylated (dashed green lines in Figure 6A) configurations. Several general trends emerge: first, irrespective of hydroxylation, the formation of V_Mg_H_2_ defects is most favored at low pH, yielding very low or even negative surface energies under strongly acidic conditions, which then progressively increase with pH. Second, defect formation is more favorable on more open facets, where breaking fewer Mg–O bonds incurs a lower energy cost. Finally, surface hydroxylation (green shading) has a similar stabilizing effect on all defective facets, due to the presence of low-coordinated anions that facilitate water dissociation.

Beyond these common trends, the absolute values of surface energies and the relative stability of ideal stoichiometric versus V_Mg_H_2_-containing terminations remain strongly orientation-dependent. On the (100) facets, defective terminations are clearly favored over ideal ones across the entire pH range, irrespective of hydroxylation. Similarly, for the pristine (110) and (111) surfaces, defective terminations dominate across the entire pH range (red and green solid lines). In contrast, upon hydroxylation the gap between the two narrows down: while defective and ideal terminations exhibit quite distinct surface energies under acidic conditions, they converge to similar values at high pH (red and green dashed lines). This behavior reflects a weaker thermodynamic driving force for defect formation in basic environments enhanced by the protective effect of surface hydroxylation.

### Mg Extraction

Before discussing the resulting pH-dependent surface behavior, we complement these thermodynamic results with insights into the energy profiles of Mg^2+^ extraction from the three facets, which also show a well-pronounced orientation dependence. As expected from the number of Mg–O bonds broken upon extraction, the required energy cost is the highest on the close-packed (100) surface (3.9 eV), Figure 6B, and decreases markedly on the more open (110) and (111) facets (2.1 and 1.7 eV, respectively). Although these energy differences are obtained for pristine terminations, neglect the stabilizing influence of water environment, and thus do not represent actual dissolution barriers, they effectively capture the underlying bond strengths and resulting orientation dependence.

## Discussion

In the following discussion we will focus on the calculated thermodynamically favored hydroxylated V_Mg_H_2_-containing terminations, delineate the pH-dependent behavior of the three facets and connect these findings to the experimental observations.

Most notably, the negative and/or very small surface energies calculated at acidic pH across all orientations align with the observed rapid Mg^2+^ release and the concomitant initial pH increase under acidic and neutral conditions. We note that, despite a strong thermodynamic driving force, surface dissolution is anticipated to proceed much more slowly on (100) facets due to their highest Mg^2+^ extraction barrier. Regarding the (111) facets, they are likely to contribute only marginally to the initial rapid dissolution regime, as they are largely absent in the original cubic MgO particles. Finally, given the rapid pH increase, an equilibrium/stationary particle shape is unlikely to develop in this initial regime, during the short time these pH conditions persist in experiments.

The progressive increase of calculated defective surface energies with increasing pH reveals the diminishing thermodynamic driving force for surface dissolution. This behavior is consistent with the pronounced deceleration observed experimentally, which can therefore be ascribed to the rise in pH. This trend is particularly evident for the (110) and (111) terminations, where the surface energies of hydroxylated terminations, whether defective or not, become virtually equal at high pH. However, in contrast to (110)-oriented facets, the energies of (111) terminations remain negative and very low up to relatively high pH values, suggesting that their slow dissolution may continue also under more basic conditions, consistently with the observed emergence of ultimate truncated octahedral shapes. For the (100) facet, the high density of surface V_Mg_H_2_ defects used in the calculations likely overestimates their actual surface concentration, which is kinetically limited by the least favorable Mg^2+^ extraction barrier from this compact termination. Kinetic effects will also influence the (110) and (111) terminations, albeit likely to a somewhat lesser extent.

We also notice that at high pH, the formation of Mg­(OH)_2_ becomes thermodynamically favorable. Given solubility product constant *K*_sp_ ≈ 10^–11^ for Mg­(OH)_2_ at room temperature, even modest Mg^2+^ release upon dissolution (∼10^–4^–10^–3^ M; consistent with observed particle shrinkage over 60 min) exceeds brucite solubility, rendering the solution supersaturated. The consequent brucite precipitation consumes the Mg^2+^ from ongoing MgO dissolution, thereby stabilizing the pH (quasi-stationary regime). The balance between ongoing MgO dissolution, Mg^2+^ release, and brucite nucleation/growth quantitatively accounts for the pH in the quasi-stationary regime, particularly under conditions where hydrodynamic mixing limits local supersaturation. As a reference, we indicate the calculated pH range for bulk brucite formation in Figure 6A, which extends from about 15.3 ([Mg^2+^] = 10^–7^ M) and 14.3 ([Mg^2+^] = 10^–5^ M) down to 12.6 ([Mg^2+^] = 0.025 M, corresponding to all MgO being dissociated), and covers slightly higher pH values compared to experimental estimations. Two mechanisms of Mg­(OH)_2_ formation can be envisaged: either precipitation from solution or direct formation at facets of MgO particles. While mostly freestanding brucite particles are observed in the experiments, formation of thin brucite overlayers at MgO facets cannot be entirely excluded. The present calculations provide evidence for the stability of such surface brucite. Indeed, increasing coverage of protonated Mg vacancies formed upon dissolution in the surface layer progressively transforms it into a brucite-like compound. Specifically, in the (2 × 2) surface unit cell used in the calculations, replacing two Mg^2+^ cations with four protons yields an overall composition of two Mg­(OH)_2_, which can be regarded as an epitaxial brucite overlayer on an ideal MgO termination. We find that, regardless the surface orientation, the energy of brucite in such overlayers is systematically more favorable than in bulk brucite, suggesting that formation of such low dimensional brucite phase on MgO surfaces is favored over 3D-like brucite structures – either freestanding or in contact with MgO. This enhanced stability of the overlayer arises primarily from the Mg–O bonds between the overlayer and the MgO support, and from its ultrathin nature, which enables efficient accommodation of the lattice mismatch by the brucite overlayer. However, since such structural flexibility is readily lost in thicker films, we anticipate that stabilization of low dimensional brucite on MgO is limited to small thicknesses only. This brucite overlayers can partly passivate the surface, thereby slowing down MgO dissolution.

Finally, the measured stabilized pH value of about 12, together with the corresponding quasi-stationary shapes of MgO particles over extended experimental time scales are consistent with the emergence of stationary/equilibrium MgO morphologies at high pH. Regarding the actual estimation of particle shape from the energies of the most stable hydroxylated defective configurations, in absence of precise information on orientation-resolved dissolution kinetics and the resulting defect densities, any attempt to quantitatively match the experimental data remains inherently arbitrary. However, it is worth noticing that the calculated similarity of surface energies of hydroxylated terminations—whether defective or not—at high pH supports the approximation employed in ref 59, where Wulff shapes were successfully estimated using only the surface energies of hydroxylated stoichiometric terminations, despite the idealized nature of the underlying assumption.

## Conclusion

Using TEM, we have investigated the time-dependent morphological evolution of MgO smoke cubes in aqueous environments while simultaneously monitoring the solution pH. When dissolution is initiated in distilled water (pH ≈ 6.4), two distinct regimes are observed. The initial stage, lasting only minutes, features a rapid pH rise to 11–12, correlating with the complete dissolution of the smallest particles and the onset of (110)-oriented faceting on larger MgO cubes. This process accelerates under more acidic conditions but is entirely suppressed when dissolution begins in an alkaline medium (pH ≈ 11). Subsequently, the system enters a quasi-stationary regime in which the pH remains stable at 11–12 over several days, brucite forms progressively in solution, and the dissolution and morphological evolution of MgO significantly slow, exposing larger particles with well-defined truncated cubic or octahedral shapes.

To rationalize these behaviors, we employed models of low-index MgO surfaces incorporating point defects expected at H^+^-rich terminations (pH < PZC). Our calculations indicate a strong thermodynamic driving force promoting the formation of such dissolution-related defects across a broad pH range from acidic to neutral, largely independent of surface orientation. This process, however, is modulated by kinetic limitations — particularly the high energy barrier of Mg extraction from the dense (100) surface. At higher pH (∼12), the driving force diminishes considerably, as surface hydroxylation renders the energies of defective and defect-free terminations nearly equivalent. These stable, high-pH conditions favor the development of quasi-stationary or near-equilibrium MgO particle shapes, consistent with experimental observations. While surface energies of ideal hydroxylated facets provide a useful first approximation, a quantitatively robust understanding will require detailed, orientation-resolved characterization of surface defect types and densities. Our study provides insights into water interaction with ionic oxides under realistic aqueous conditions, establishing general principles for controlling dissolution, morphology evolution, and passivation—critical not only for MgO but also for other ionic oxide nanoparticles whose self-adjusted local pH and defect chemistry govern longevity, reactivity, and transformation pathways.
